# Artificial Intelligence Integrated with Intraoral Digital Imaging in Dental Caries Detection, Treatment Planning, and Clinical Decision-Making: A Scoping Review

**DOI:** 10.12688/f1000research.172671.2

**Published:** 2026-03-05

**Authors:** Sarah Kayali, Ali Golkari, Manu Mathur

**Affiliations:** 1 Institute of Dentistry, Queen Mary University of London, London, England, E1 2AH, UK

**Keywords:** Artificial Intelligence, Computer-Assisted Image Interpretation, Dental Caries, Dental Radiography, Diagnostic Imaging, Treatment Planning, Clinical Decision-making, Clinical Decision Support System

## Abstract

**Background:**

The emergence of AI technologies has revolutionised dentistry, with intraoral imaging being a key area for innovation. Despite advances and growing interest in applying AI algorithms to intraoral x-rays, the methodological quality, diagnostic validity, and clinical applicability of existing studies remain unclear.

**Aim:**

To synthesise and critically appraise the current evidence on AI integrated with intraoral digital radiographic imaging for detecting dental caries in adults, focusing on diagnostic accuracy compared with gold-standard methods and examining methodological quality, clinical applicability, and implementation challenges.

**Methods:**

Following the JBI scoping review framework and PRISMA-ScR reporting guidelines, a comprehensive literature search was conducted across the PubMed, Scopus, and IEEE Xplore databases from January 2015 to May 2025. Studies that met the predefined eligibility criteria were included. Thematic analysis, combining inductive and deductive approaches following Braun and Clarke’s framework, identified five themes. The CASP quality appraisal was performed to ensure methodological rigour.

**Results:**

Ten peer-reviewed studies were included in the final data analysis. AI systems detected a greater number of carious lesions than human clinicians, particularly in early-stage caries, with representative metrics including 88% sensitivity, 91% specificity, and 89% accuracy. Other models reported F1-scores up to 89% and AUC ≈95%. Methodological diversity was notable, with histology-validated designs providing the strongest evidence. Implementation challenges included limited external and real-world validation, clinician oversight, ethical/regulatory considerations, and inadequate model interpretability.

**Conclusion:**

AI exhibits strong potential to enhance early caries detection on intraoral radiographs and support clinical decision-making in adults. Fully realising AI’s clinical potential requires overcoming implementation and methodological challenges. Standardised validation methods across diverse populations and settings are crucial to ensure AI diagnostic reliability and generalisability. Current AI applications in dentistry are primarily designed to assist clinicians in detecting caries; however, their greatest potential lies in a future where they can independently guide treatment planning decisions.

## 1. Introduction

Proper and timely detection of dental caries, along with appropriate decision-making on whether to intervene, use preventive measures, or monitor, is critical for maintaining oral health and preventing disease progression. It is the necessary first step of the process to preserve natural tooth structure, reduce the need for invasive and costly treatments, and minimise complications such as pain, infection, and abscesses. ^1– 3^ From a public health perspective, accurate detection and sound treatment planning reduce the financial burden on healthcare systems by minimising the need for complex interventions. At the individual level, they support long-term function, improved quality of life, and alignment with the principles of minimally invasive dentistry. ^1, 3– 5^

Missed or incorrect diagnoses can lead to both over-treatment and under-treatment, with consequences for patient well-being, imposed costs, healthcare resources, and clinician liability. ^6, 7^ Delayed or inappropriate referrals increase the likelihood of advanced disease requiring more invasive and costly treatments, while unnecessary referrals can expose patients to repeat diagnostic procedures, additional costs, and radiation exposure. ^8– 10^ Improving diagnostic precision is therefore crucial for both patient care and system-level efficiency.

Current diagnostic methods, which include the combination of visual-tactile examinations and viewing two-dimensional radiographs, face well-recognised limitations. ^11– 13^ While more sophisticated techniques, such as 3-D scans, are not practically appropriate for general dental practice use. ^14^ As a result, artificial intelligence (AI) has emerged as an attractive solution. Machine learning and deep learning algorithms have shown the ability to analyse intraoral radiographs with high accuracy. ^15, 16^ Intraoral radiographs, such as bitewing and periapical, have been a key focus, with AI systems demonstrating strong diagnostic metrics—high sensitivity, specificity, and AUC values—while also reducing the time required for interpretation. ^17– 19^

Integrating AI into dental diagnostics offers multiple benefits that extend beyond improved accuracy. AI systems consistently demonstrate higher sensitivity for subtle lesions, provide real-time decision support, and reduce inter- and intra-observer variability. ^16, 20^ They also classify lesion severity, align with frameworks such as ICDAS or ICCMS, and contribute to evidence-based treatment planning. Significantly, automated analysis improves efficiency by reducing interpretation time and freeing clinicians to focus on patient communication and holistic care. ^21, 22^

### 1.1 Rationale for review and gap in the literature

The rapid advancement of AI in dental diagnostics has led to a growing body of literature on its application for caries detection from intraoral radiographs. However, the evidence remains fragmented and methodologically heterogeneous. Studies vary widely in imaging modalities, dataset quality, annotation protocols, lesion definitions, and AI architectures. Many rely on small, single-centre samples, internal cross-validation, and non-standardised outcome reporting, with limited attention to calibration, decision thresholds, or model interpretability. Such methodological heterogeneity restricts comparability and reduces confidence in generalisability. ^23– 25^

Most, if not all, of the literature focuses on the increased number of caries detection when AI is integrated. Limited attention has been given to whether AI might lead to over-detection of lesions and, consequently, unnecessary treatment. It also remains unclear whether existing studies have adequately addressed external validity through independent, multicentre validation or considered the ethical, legal, and regulatory readiness of AI systems for safe clinical deployment.

### 1.2 Aims and objectives

This scoping review aims to map and synthesise the current literature on AI integrated with intraoral digital radiographic imaging for detecting dental caries in adults, with emphasis on studies comparing AI diagnostic performance against established gold-standard diagnostic methods. Specifically, this review (i) identify and summarise studies applying AI to bitewing and periapical radiographs; (ii) identify and categorise AI model types (e.g., machine learning, deep learning, convolutional and segmentation networks) and intraoral imaging systems; (iii) evaluate AI diagnostic accuracy in comparison to histology, expert clinician assessment, or validated diagnostic criteria; (iv) assess reported impact on clinical decision-making—including treatment planning, diagnostic confidence, and workflow efficiency; and (v) Identify evidence gaps and implementation challenges to guide future research priorities, clinical integration approaches, and policy for AI-assisted caries diagnostics.

## 2. Methodology

### 2.1 Study design

This study employed a scoping review methodology, guided by the Joanna Briggs Institute (JBI) framework for evidence synthesis, ^26^ and reported in accordance with the PRISMA-ScR checklist to ensure transparency and reproducibility. ^27, 50^ The JBI approach, which builds on the foundational framework of Arksey and O’Malley (2005) and is further refined by Levac et al. (2010), ^28, 29^ provides structured yet flexible guidance, making it particularly suited to broad and emerging research areas. This methodology was selected to systematically map and appraise the diverse and heterogeneous body of literature on AI applied to intraoral radiography for caries detection in adults. Unlike systematic reviews, which typically address narrowly defined effectiveness questions, scoping reviews allow the inclusion of varied study designs, methodologies, and outcomes, thereby capturing the breadth of existing research. ^30^ This approach is especially appropriate given the rapidly evolving, multidisciplinary nature of AI in dentistry, where studies differ in terms of AI models, imaging systems, validation strategies, and clinical applications.

This scoping review protocol was developed in advance in November 2024 to ensure a transparent and systematic methodological approach. It outlines the review’s aim, objectives, eligibility criteria, search strategy, study selection process, data extraction, and data analysis and synthesis. Additionally, although the protocol was not formally registered, all steps were thoroughly documented and consistently applied throughout the review process.

### 2.2 Defining the research question

The research question was developed in line with the JBI methodology and framed using the Population–Concept–Context (PCC) framework to ensure clarity and transparency. The central question guiding this review is: **“Among adults undergoing dental examination for caries (Population), what is the current evidence on the use of artificial intelligence (AI) integrated with intraoral digital imaging systems for caries detection and its impact on diagnostic accuracy, treatment planning, and clinical decision-making (Concept), in clinical or research settings using intraoral radiography (Context)?”**

**2.2.1 Eligibility criteria**

**P (Population):** Adults (≥18 years) undergoing dental examination or treatment.

**C (Concept):** Studies investigating the integration of artificial intelligence (AI), including machine learning, deep learning, or neural networks, with intraoral digital radiographic imaging (bitewing and periapical X-rays) for:

• Detection of dental caries.
• Comparison of AI diagnostic accuracy with established gold-standard methods such as histological validation, clinical visual examination or expert consensus.
• Evaluating the influence of AI on treatment planning, diagnostic confidence, and clinical decision-making.
• Diagnostic accuracy measures such as sensitivity (the model's ability to correctly identify diseased surfaces), specificity (the model's ability to correctly identify non-diseased surfaces), accuracy (the overall proportion of correct classifications), precision or positive predictive value (PPV; the proportion of true positives among all positive predictions), negative predictive value (NPV; the proportion of true negatives among all negative predictions), F1-score (the harmonic mean of precision and sensitivity), and area under the receiver operating characteristic curve (AUC; a measure of overall discriminatory ability).

**C (Context):** Clinical or research-based diagnostic settings using intraoral radiography (bitewing or periapical), and examining the application of AI in caries management, including both primary and specialist care environments.

Original diagnostic accuracy studies, comparative designs, in vivo or in vitro investigations, randomised controlled clinical trials, and reviews of these original studies were included if published in English between January 2015 and May 2025.

### 2.3 Identifying relevant studies

Relevant studies were identified through a comprehensive search of three major electronic databases: **PubMed, Scopus, and IEEE Xplore**. Search strategies were developed in consultation with an expert librarian at Queen Mary University of London (QMUL) to ensure methodological rigour and comprehensive coverage. A combination of controlled vocabulary, Medical Subject Headings (MeSH) terms for PubMed, and corresponding subject headings for Scopus, as well as free-text keywords and their synonyms, is used to cover the full scope of literature related to the research question, structured across five concepts groups: (i) artificial intelligence, (ii) intraoral digital radiography, (iii) dental caries, (iv) detection and diagnostic accuracy, and (v) clinical outcomes.

The search strategy used Boolean operators (OR, AND) to link different concept groups; (OR) connected synonymous terms within each concept, and (AND) linked different thematic areas. Truncation symbols and wildcards were also applied, with database-specific syntax tailored for each database.

Preliminary searches were piloted and iteratively refined to optimise sensitivity and specificity, with strategies re-visited. Backwards citation searching and manual hand-searching were also conducted. The retrieved records formed the basis of evidence for subsequent screening against predefined eligibility criteria.

**2.3.1 Search Strategy for PubMed**

In accordance with PRISMA-ScR guidelines and to ensure full methodological transparency and reproducibility, the verbatim PubMed search strategy is provided below. The search was conducted using the National Library of Medicine’s PubMed Advanced Search interface. An initial search was performed on 19 December 2024 to identify preliminary literature for the review, followed by a final comprehensive search in February 2025, which served as the formal search date for this scoping review. Filters were applied to include only English-language studies published within the past ten years, ensuring relevance to recent advancements, evidence-based practices, and emerging developments in AI relevant to contemporary clinical and research contexts. The search covered all eligible literature indexed in PubMed from database inception to February 2025. A supplementary manual search was conducted in May 2025 to identify newly published studies. All results were exported immediately to ensure accuracy and reproducibility. The detailed search string below reflects the final query executed in the database, including all Boolean operators, keywords, and controlled vocabulary terms used during the literature search.

The complete PubMed search strategy:

((((((((((((("Artificial Intelligence"[Mesh]) OR (artificial intelligence)) OR (machine learning)) OR (deep learning)) OR ("Neural Networks, Computer"[Mesh])) OR ("Convolutional Neural Networks"[Mesh])) OR (automated detection)) OR (pattern recognition)) OR (computer aided detection)) OR (automated intelligence)) AND (((((((((((intraoral radiograph) OR (intraoral x-ray)) OR (periapical radiograph*)) OR (bitewing radiograph*)) OR ("X-Ray Film"[Mesh])) OR ("Radiography, Dental, Digital"[Mesh])) OR ("Photography, Dental"[Mesh])) OR ("Diagnostic Imaging"[Mesh])) OR (Image*)) OR (Radio*)) OR (Photo*))) AND (((("Dental Caries"[Mesh]) OR (caries)) OR ("Tooth Demineralisation"[Mesh])) OR (tooth demineral*))) AND (((((("Diagnosis"[Mesh]) OR (detection)) OR (detect*)) OR (screening)) OR (image reading)) OR (lesion identification))) AND ((((((((("Therapeutics"[Mesh]) OR (treatment planning)) OR (treatment outcomes)) OR (clinical decision making)) OR (clinical outcomes)) OR (intervention)) OR (management)) OR (precision medicine)) OR (risk assessment)) Filters: in the last 10 years, English

Ultimately, the complete search strategies for all three databases are provided in the supplementary materials, accessible via the external data repository, ensuring full transparency and reproducibility of the search process. ^50^ Please see below under data availability.

### 2.4 Study selection

The selection of studies was conducted systematically and transparently, in line with predefined eligibility criteria, to ensure the inclusion of only relevant, high-quality evidence in data extraction and subsequent phases. At each stage, screening decisions were documented using a standardised form, with reasons for exclusion recorded to maintain transparency and reproducibility.

**2.4.1 Selection Step 1: Identifying duplicates**

After completing the database searches, all identified publications were imported into EndNote 21 (Clarivate, USA, 2023) reference management software. ^31^ The software’s automatic deduplication feature was initially used to remove duplicate records. The remaining references were exported from EndNote into the Rayyan screening AI tool (Rayyan Systems Inc., Qatar, 2025). ^32^ This tool automatically identified additional potential duplicates and flagged them, letting the researchers to choose the most complete or appropriate version and delete the other(s).

**2.4.2 Selection Step 2: Title and abstract screening**

A thorough, systematic screening of the titles and abstracts of all records was conducted independently by two researchers (SK, AG) using the Rayyan AI tool, against the predefined eligibility criteria. Studies that clearly indicated the relevance of AI integration with intraoral imaging for caries detection were retained, while those focused on unrelated topics were excluded. Additionally, ambiguous titles or abstracts that lacked sufficient clarity were retained for further assessment at the subsequent stage. Any discrepancies at this stage were resolved through consensus discussions among the researchers to maintain methodological rigour and ensure robust decision-making.

**2.4.3 Selection Step 3: Critical appraisal**

The full texts of all remaining studies were obtained. Where access was restricted, requests were made through QMUL library services. Each study was then critically appraised using the Critical Appraisal Skills Programme (CASP) checklist appropriate to its study design. The critical appraisal was conducted independently by GPT-4o Team (OpenAI, USA, 2025) and one researcher (SK), both of whom were trained and calibrated by the co-author (AG). A standardised reporting format was used, comprising a study summary, CASP scoring table, and overall recommendation. Discrepancies in reviewer judgments were resolved by the co-author (AG) to ensure consistency and methodological rigour.

**Table 1.** Breakdown of reasons and numbers of studies excluded during title and abstract screening and full text assessment.

Initial title and abstract screening stage/No. of studies	Full-text screening stage/No. of studies
**Reason 1:** Studies used extraoral radiographic imaging systems (n = 46)	**Reason 1:** Studies lacking the proper use of the gold standard (n = 40)
**Reason 2:** Studies used intraoral photographs or intraoral scanners (n = 38)	**Reason 2:** Studies lacking inter-rater reliability assessment and calibration standards (n = 16)
**Reason 3:** Studies applied AI in other fields of dentistry (n = 184)	**Reason 3:** Methodological limitations; AI reliability, heterogeneity and validation Issues (n = 10)
**Reason 4:** Studies focused on paediatric population (n = 20)	**Reason 4:** CASP quality appraisal (n = 5)
**Total n = 288**	**Total n = 71**

**Table 2:** Summary of the CASP appraisal quality assessment for Diagnostic accuracy studies.

CASP factors	*Rodrigues et al.*	*Klein et al.*	*Ying et al.*	*Boldt et al.*	*Liu et al.*	*Chen et al.*	*Lin et al.*
1. Did the study address a clearly formulated research question?	Yes	Yes	Yes	Yes	Yes	Yes	Yes
2. Was there a comparison with an appropriate reference standard?	Yes	Yes	Yes	Yes	Yes	Yes	Yes
3. Did all patients get the diagnostic test and reference standard?	Yes	Yes	Yes	Yes	Yes	Yes	Yes
4. Could the results of the test have been influenced by the results of the reference standard?	No	No	No	No	No	No	No
5. Is the disease status of the tested population clearly described?	Yes	Yes	Yes	Yes	Yes	Yes	Yes
6. Were the methods for performing the test described in sufficient detail?	Yes	Yes	Yes	Yes	Yes	Yes	Yes
7. What are the results?	Yes	Yes	Yes	Yes	Yes	Yes	Yes
8. How sure are we about the results? Consequences and cost of alternatives performed?	Yes	Yes	Yes	Yes	Yes	Yes	Yes
9. Can the results be applied to your patients/the population of interest?	Yes	Yes	Yes	Yes	Yes	Yes	Yes
10. Can the test be applied to your patient or population of interest?	Yes	Partially	Partially	Yes	Yes	Yes	Yes
11. Were all outcomes important to the individual or population considered?	Yes	Yes	Yes	Yes	Yes	Yes	Yes
12. What would be the impact of using this test on your patients/population?	Support early detection and minimise invasive treatment, especially with potential improvement of AI models.	Findings will influence annotation practices in AI research and improve data quality for training diagnostic models.	Supports the clinical use of YOLOv5 and Trans-UNet for caries detection; networks showed performance comparable to dentists.	Establishing standardised gold standard enhances reliability and transparency of AI diagnostics, leading to improved diagnostic accuracy and patient care in dentistry.	The tool would improve early caries detection accuracy, enhance clinical decision-making, and potentially decrease unnecessary treatments.	Could significantly enhance early detection of proximal caries, potentially leading to more timely preventive interventions and improved oral health outcomes.	EE strategy could improve sensitivity in detecting early proximal caries, aiding non-invasive management and treatment planning.

**Table 3:** Summary of the CASP appraisal quality assessment for the RCT studies.

CASP factors	Das et al.	Devlin et al.
1. Did the study address a clearly formulated research question?	Yes	Yes
2. Was the assignment of participants to interventions randomised?	Yes	Can’t Tell
3. Were all participants who entered the study accounted for at its conclusion?	Yes	Yes
4. (a) Were the participants ‘blind’ to intervention they were given?	Can’t Tell	Yes
4. (b) Were the investigators ‘blind’ to the intervention they were giving to participants?	No	Yes
4. (c) Were the people assessing/analysing outcome/s ‘blinded’?	No	Yes
5. Were the study groups similar at the start of the randomised controlled trial?	Yes	Yes
6. Apart from the experimental intervention, did each study group receive the same level of care (that is, were they treated equally)?	Yes	Yes
7. Were the effects of intervention reported comprehensively?	Yes	Yes
8. Was the precision of the estimate of the intervention or treatment effect reported?	Yes	Yes
9. Do the benefits of the experimental intervention outweigh the harms and costs?	Yes	Can’t Tell
10. Can the results be applied to your local population/in your context?	Yes	Yes
11. Would the experimental intervention provide greater value to the people in your care than any of the existing interventions?	Yes	Yes

**Table 4:** Summary of the CASP appraisal quality assessment for the cross-sectional study.

CASP factors	García-Cañas et al.
1. Did the study address a clearly focused issue?	Yes
2. Did the authors use an appropriate method to answer their question?	Yes
3. Were the subjects recruited in an acceptable way?	Yes
4. Were the measures accurately measured to reduce bias?	Yes
5. Were the data collected in a way that addressed the research issue?	Yes
6. Did the study have enough participants to minimise the play of chance?	Yes
7. How are the results presented, and what is the main result?	Yes
8. Was the data analysis sufficiently rigorous?	Yes
9. Is there a clear statement of findings?	Yes
10. Can the results be applied to the local population?	Yes
11. How valuable is the research?	Yes

**Table 5.** Summary of main characteristics of the included studies.

ID	Title of the paper	Authors/Year of publication	Journal	Study design	Country	Sample size	Imaging system	AI methodology
**1**	Accuracy Assessment of Human and AI-Assisted Bitewing Radiography and NIRI-Based Methods for Interproximal Caries Detection: A Histological Validation	Rodrigues et al., 2025 ^34^	Caries Research	In-vitro diagnostic accuracy study	Spain	A total of 171 proximal surfaces from 100 extracted posterior teeth	Intraoral Bitewing radiographs and NIRI intraoral scans	AI-assisted bitewing radiography assessment using a Deep CNN-based software Denti.AI; an AI model integrated with a radiographic interpretation tool
**2**	From inconsistent annotations to ground truth: aggregation strategies for annotations of proximal carious lesions in dental imagery	Klein et al., 2025 ^35^	Journal of Dentistry	In-vitro diagnostic performance evaluation study	Germany and the Czech Republic	A total of 1007 proximal surfaces from 522 extracted posterior teeth	Orthoradial radiographs and Near-Infrared Light Transillumination (NILT)	Evaluation of annotation aggregation strategies: Majority Voting (MV), Weighted Majority Voting (WMV), Dawid-Skene (DS), Multi-Annotator Competence Estimation (MACE)
**3**	Performance comparison of multifarious deep networks on caries detection with tooth X-ray images	Ying et al., 2024 ^36^	Journal of Dentistry	Comparative diagnostic accuracy study	China	A total of 392 periapical radiographs (346 training and validation dataset, 46 testing dataset); 135 teeth in the testing dataset	Periapical digital radiographs	Four deep networks types: 1. YOLOv5 and DETR object detection networks. 2. UNet and Trans-UNet segmentation networks
**4**	Developing the Benchmark: Establishing a Gold Standard for the Evaluation of AI Caries Diagnostics	Boldt et al., 2024 ^37^	Journal of Clinical Medicine	In vitro diagnostic accuracy study	Germany	A total of 1071 bitewing radiographs from 179 extracted permanent human teeth	Standardised bitewing radiographs using the parallel technique	Evaluation of the performance of an AI algorithm model against a histology-based gold standard benchmark
**5**	Evaluating the Accuracy of AI-Based Software vs Human Interpretation in the Diagnosis of Dental Caries Using Intraoral Radiographs: An RCT	Das et al., 2024 ^38^	Journal of Pharmacy and Bioallied Sciences	Randomised controlled trial (RCT)	India and Saudi Arabia	200 intraoral radiographs were obtained from patients aged 18 to 65 years seeking dental care	Two bitewings and two periapical radiographs per participant using digital intraoral X-ray equipment; anonymised and standardised radiographs collected prospectively	Deep learning-based AI software to detect carious lesions on intraoral radiographs
**6**	Artificial intelligence for caries detection: a novel diagnostic tool using deep learning algorithms	Liu et al., 2024 ^39^	Oral Radiology	Diagnostic accuracy study using deep learning	China	4278 periapical radiographs (12,524 single-tooth images)	Digital periapical radiographs from clinical settings	ResNet-based CNN with Segment Anything Model (SAM); integrated Grad-CAM for visual support
**7**	Diagnosis of Interproximal Caries Lesions in Bitewing Radiographs Using a Deep Convolutional Neural Network-Based Software	García-Cañas et al., 2022 ^40^	Caries Research	Analytical, observational, and cross-sectional study	Spain	300 digital bitewing radiographs of posterior teeth taken from 150 patients aged 16-85 years	Digital bitewing radiographs	Deep CNN-Based Software (Denti.Ai) with different caries detection thresholds (Model 1 to Model 4)
**8**	Detecting Proximal Caries on Periapical Radiographs Using Convolutional Neural Networks with Different Training Strategies on Small Datasets	Lin et al., 2022 ^41^	Diagnostics	Diagnostic accuracy study	China	800 periapical radiographs (600 training/validation, 200 testing) from 3165 initial periapical radiographs taken from 385 men and 415 women (mean age: 45.3 years)	Periapical radiographs (BMP format) from PACS system, acquired via the paralleling technique	Pretrained Cifar-10Net CNN with three training strategies: IR (image recognition), EE (edge extraction), IS (image segmentation); trained using transfer learning and fine-tuning
**9**	Detection of Proximal Caries Lesions on Bitewing Radiographs Using Deep Learning Method	Chen et al., 2022 ^42^	Caries Research	Diagnostic accuracy study	China	978 bitewing radiographs;10,899 proximal surfaces analysed	Digital bitewing radiographs	Faster R-CNN deep learning object detection framework for caries localisation and classification
**10**	The ADEPT study: a comparative study of dentists’ ability to detect enamel-only proximal caries in bitewing radiographs with and without the use of AssistDent artificial intelligence software	Devlin et al., 2021 ^43^	British Dental Journal	RCT-Comparative diagnostic accuracy study	United Kingdom	24-bitewing radiographs.23 dentists (11 in the control group and 12 in the experimental group)	Digital bitewing radiographs	AssistDent AI software (machine learning algorithm)

ID	Dental focus	Dental setting	Diagnostic accuracy measures	Gold standard	Validation	Key findings	Limitations and Bias
**1**	Interproximal caries detection	Laboratory research setting (in vitro); posterior teeth collected and preserved for scanning and histology evaluation	**AI guided radiographic assessment:** Se = 13.7%, Sp = 95.9%, PPV = 71%, NPV = 59.8%, F1 = 23%, AUC = 0.548 **Examiners radiographic assessment:** Se = 52%, Sp = 84.6%, PPV = 71.6%, NPV = 70.3%, F1 = 60%, AUC = 0.684, K = 0.459	Histological validation using optical microscopy evaluation	All methods validated against histology; Fleiss Kappa for examiner agreement; statistical comparisons via Chi-Square, McNemar, and Wilcoxon tests	Human examiners’ radiographic assessments demonstrated high constant accuracy and superior early caries detection capabilities compared to the AI programme	1. In-vitro design may not fully replicate the clinical environment 2. Early-stage caries lesions (E1) were overrepresented in the sample, which may have affected the findings 3. Small dataset for generalisation
**2**	Primary proximal carious lesions	Laboratory research setting (in vitro), using extracted human premolars and molars teeth in a simulated clinical setup	AUROC, sensitivity, specificity, and F1-score across strategies and lesion depths (sound, enamel, dentin)	Histological examination of sectioned teeth to assess the presence and depth of carious lesions	Compared against histology as the gold standard, stratified analysis by imaging modality and lesion depth	For radiographs, MACE outperformed other strategies in unimodal datasets; DS was best in multimodal datasets; MV often underperformed across all lesion depths	1. Limited to in vitro settings may lack the variability and complexity of real-world clinical imagery 2. Potential dataset imbalance, and no feasibility discussion for clinical integration
**3**	Proximal and multifaceted caries	Clinical dental practice	**YOLOv5:** Sensitivity 82%, Specificity 94%, Percision 93%, F1-score 0.87. **Trans-UNet:** Sensitivity 81%, Specificity 92% **DETR:** Sensitivity 72%, Specificity 96%, Precision 95%, **UNet:** Sensitivity 76%, Specificity 88%, Percision 85% **Dentist:** Sensitivity 89%, Specificity 91%, Precision 91%	Expert annotations and clinical validation by senior stomatologists	Internal validation against expert annotations and clinical examination	1. YOLOv5 outperformed other networks with the highest sensitivity, specificity, F1-score, and Youden index 2. No statistically significant differences between deep networks and between well-trained networks and dentists in caries detection	1. Only periapical radiographs were used with a small single-institution dataset 2. Potential for bias in clinical application 3. The performance and experience of the dentists included are not representative 4. Limited practical feasibility discussion
**4**	Detection and staging of proximal caries: provide a validated framework for AI model benchmarking	Simulated clinical setting with high-resolution extracted human teeth datasets	Sensitivity: 0.565, Specificity: 0.956, Accuracy: 0.799, AUC: 76.1, MCC: 0.578, F1-score: 0.693	Histological validation of each lesion based on thin-section microscopy, compared with examiner ratings	Internal validation using blinded human examiners and statistical analysis (ICC = 0.993)	High inter-examiner agreement; the dataset offers realistic lesion representations and robust histological reference standards	Only one imaging system and technique were used, which lacks diversity in patient demographics. The focus was on benchmarking rather than real-time AI testing
**5**	Detection of dental caries (proximal and general); supports diagnostic decision-making	Clinical dental care setting	**AI:** Sensitivity 88%, Specificity 91%, Accuracy 89% **Human:** Sensitivity 84%, Specificity 88%, Accuracy 86%	Consensus diagnosis from two experienced dental radiologists (blinded to AI and each other’s assessments)	Internal validation using statistical comparison against predefined benchmark values (85/90/88%); no external dataset	AI outperformed human interpretation in sensitivity, specificity, and accuracy, exceeding benchmark values	AI software model specifics undisclosed; limited to intraoral radiographs; benchmark-based comparison only
**6**	1. Detection of dental caries on single-tooth periapical radiographs 2. supports clinical decision-making and workflow	Clinical hospital dental imaging repository with expert-annotated cases	Accuracy: 0.885, Sensitivity: 0.894, Specificity: 0.887, F1-score: 0.886, AUC: 0.954 (all with 95% CI)	Expert clinical diagnosis from dental records and ICDAS-coded manual review by specialists	Internal validation; Cohen’s and Fleiss’ kappa for inter-rater agreement; Grad-CAM and overlay visualisation used	AI Model achieved high diagnostic accuracy; visual interpretation support via Grad-CAM enhanced user confidence	1. Cropping tilted teeth still requires manual image rotation, limiting full automation. 2. Diagnoses were subjective and dependent on dentists’ clinical experience, which may affect the reliability of the training dataset
**7**	1. Detection of interproximal caries lesions 2. Clinical support in early caries identification	Private dental clinic	**Best model (Model 2):** Accuracy 82%, Sensitivity 69.8%, Specificity 85.4%, AUC 0.777 (95% CI 0.729–0.824)	Clinical-visual examination, radiographic inspection, and/or cavity opening for dentin caries validated by two experienced dentists	Internal validation with ROC and AUC analyses; confidence intervals reported for all major metrics	AI software demonstrated acceptable diagnostic performance, particularly at moderate lesion thresholds (≥25% probability)	No external multicenter validation; focused mainly on interproximal lesions; dependence on predefined thresholds
**8**	Detection of proximal caries on posterior teeth using periapical radiographs	Hospital of Stomatology, Fujian Medical University (clinical image source); lab-based CNN development	AUC: EE = 0.860, IR = 0.805, IS = 0.549; Accuracy: EE = 85.9%, IR = 82.1%, IS = 60.6%; Sensitivity: EE = 86.9%, Specificity: EE = 85.2%, F1-score: EE = 0.837	Consensus annotation by three endodontists; test dataset evaluated according to predefined evaluation criteria, which were used to compare the performance of IR, IS, EE and human observers	Internal validation using a separate test set; statistical comparisons (Z-test, chi-square) with 95% CI; comparison with human observer consensus	The edge extraction (EE) strategy significantly outperformed others and human eyes in detecting both enamel and dentin lesions. EE achieved the highest AUC, F1-score, and sensitivity	Lack of standardisation of the radiological dosage.; small sample size; no clinical outcome data; no external or cross-institution validation; IS strategy underperformed
**9**	Detection of proximal caries lesions: early, moderate, and advanced stage differentiation	Clinical imaging dataset from a university school and hospital of stomatology in Beijing, China	**AI Model:** Accuracy 87%, Sensitivity 72%, Specificity 93%, F1-score 0.74 **Students:** Accuracy 82%, Sensitivity 47%, Specificity 94%, F1-score 0.57	Annotations by two endodontists and a radiologist based on clinical and radiographic criteria	Statistical significance tested via McNemar’s test; ROC curves analysed; p < 0.001	Faster R-CNN significantly outperformed postgraduate students in detecting proximal caries, especially in early lesions	Limited to comparison against student raters; not tested against highly experienced clinicians; internal dataset only
**10**	Enamel-only proximal caries detection	General dental practice and dental teaching hospital	Sensitivity: 75.8% with AI vs. 44.3% without AI; Specificity: 85.4% with AI vs. 96.3% without AI	Expert panel annotations (at least three independent dento-maxillofacial radiologists/professors)	Internal validation with an expert panel	Use of AssistDent significantly improved sensitivity (71% increase) while slightly reducing specificity (11% decrease). Significant improvement (p < 0.01)	Feasibility, practicality, and cost-effectiveness of AI are not discussed; there is a higher false-positive rate with AI use

ID	Conclusion	Recommendation
**1**	1. AI models require further refinement for higher early lesion sensitivity improvement 2. NIRI may be a promising adjunct to radiography	1. There is a need to modify the current diagnostic criteria for AI programmes to allow for early caries detection 2. Future research to optimise the digital methods to ensure their effectiveness and reliability in clinical dental practice
**2**	Optimal aggregation strategies vary by dataset type; DS and MACE are recommended over traditional MV	Informed strategy selection is essential; future research should assess clinical feasibility and include in vivo datasets
**3**	Deep networks demonstrate comparable diagnostic performance to that of experienced dentists and show promising potential clinical applications, with YOLOv5 recommended due to its superior metrics	1. Further research is recommended for practical implementation across diverse clinical settings 2. Future research is needed using more advanced deep networks, in collaboration with dentists across diverse hospitals and institutes, to broaden the generalisability of the findings
**4**	Provides a standardised dataset and gold standard for future AI benchmarking in caries diagnostics	Encourages researchers to adopt standardised databases and protocols for AI validation and clinical performance comparison against an established histology-based gold standard
**5**	AI demonstrated higher diagnostic accuracy than expert interpretation, making it a promising second opinion or adjunctive tool	Support for AI implementation in caries detection is warranted, with future research required to enhance model transparency and facilitate external validation
**6**	ResNet + SAM system effectively identifies caries in periapical images with high performance and supports visual interpretability	Encourages clinical integration of deep learning as an assessment tool for clinical decision-making and future external validation studies for general use in caries diagnostics
**7**	AI software can assist in detecting interproximal caries lesions and may complement clinical evaluation in practice	Further studies should explore AI integration across broader caries types and validate across diverse clinical settings
**8**	Preprocessing via EE significantly improves CNN detection performance, even on small datasets. Therefore, the proposed method should be regarded as a computer-aided caries detection system in clinical practice, considering its application and generalisability	Further research should increase the dataset size, utilise clinical comparisons, standardise radiographic parameters, and evaluate the influence of treatment decisions in real-world practice
**9**	Faster R-CNN demonstrated strong potential for assisting clinical caries detection, improving sensitivity without compromising specificity	1. Future research should involve validation against expert dentists and across multiple institutions 2. The generalisability of the AI model needs to be well-evidenced in future studies
**10**	AssistDent AI significantly enhances the detection of enamel-only proximal caries, which is beneficial for preventive dentistry, despite a slight decrease in specificity	AI software is recommended as a supportive diagnostic tool in general dental practice for preventive dentistry, but further developments could include monitoring the progression of caries

**Table 6.** Theme distribution: AI and intraoral imaging in dental caries detection studies.

Theme	Supporting studies ID	Total no. of studies	Main outcomes
**Theme 1:** AI Effectiveness: Diagnostic Accuracy and Comparison with the Gold Standard	1-10	10	AI demonstrated comparable or superior accuracy in caries detection compared to clinicians, especially for early-stage lesions, with higher F1 and sensitivity scores. Its performance was validated against the gold standard, including histological validation, clinical visual examination, and expert panel consensus annotations.
**Theme 2:** Clinical Implications and Relevance: AI as a Clinical Decision Support Tool, Impact on Treatment Planning	5, 6, 7, 8, 10	5	AI enhanced clinicians' sensitivity and diagnostic confidence, particularly in early caries detection; served as a clinical support decision-making tool and treatment planning aid without replacing clinician judgment. Significant positive impact on preventive and minimally invasive treatment planning, workflow efficiency, and patient communication.
**Theme 3:** Imaging Modalities and Diagnostic Variation by Radiograph Type and Lesion Severity	1, 3, 6, 7, 8, 9, 10	7	Bitewing radiographs were the most common; image quality and lesion stage significantly affected outcomes, and manual preprocessing was required in some studies. Further, performance variability was observed between bitewing and periapical radiographs.
**Theme 4:** Methodological Considerations: AI Model Strategies, Technical Design, Validation Approaches, and Limitations	2, 3, 4, 6, 7, 8, 9, 10	8	Diverse AI methodologies, such as CNNs, YOLOv5, and ResNet, were utilised; techniques like edge extraction and transfer learning enhanced performance. Robust internal validations, but were constrained by methodological issues, including single-centred studies and small datasets. The limitations included overfitting, limited external validation, challenges with clinical realism, and issues with imaging variability that affected generalisability.
**Theme 5:** Implementation Challenges, Recommendations for Practical Integration, and Future Research Directions	1-10	10	Clinicians retain diagnostic authority; however, there is a need for explainable AI tools benchmarked against a histology-based gold standard or integrated with ICDAS/ICCMS systems for the unbiased evaluation of AI-based caries detection. Practical integration barriers include transparency, cost-effectiveness, and workflow integration. A common recommendation is made for larger, longitudinal, and multicentre research studies and standardisation.

A structured scoring system was applied to the quality appraisal process, with responses coded as *Yes = 1*, *No = 0*, and *Unclear/Maybe/Not applicable = 0.5.* Studies achieving full or full-minus-one scores were rated as high quality and were included. Those with full minus two scores were rated as medium quality, with inclusion determined on a case-by-case basis depending on relevance. Studies with less than full minus two scores were considered low quality and were excluded. All CASP scores were documented with justifications for inclusion, potential inclusion, or exclusion. This dual-review approach ensured transparency, reproducibility, and accountability, while allowing flexibility to retain studies of potential value despite minor quality limitations.

**2.4.4 Selection Step 4: Full-text assessment**

The full texts of all remaining studies were independently reviewed by the researcher (SK) to assess methodological rigour, with particular attention to the use of appropriate gold-standard validation methods. Those that mixed adults with children or adolescents, lacked inter-rater reliability assessment and calibration standards, lacked clinical or external validation, and had a high or unclear risk of bias were excluded. Conference proceedings and Duplicate records of the same studies with overlapping datasets were also excluded. Ambiguous cases were discussed in detail with the co-author (AG) until consensus was reached. All studies agreed upon through this structured process formed the final sample for data extraction and synthesis. The review process was documented in a standardised Excel spreadsheet, recording reasons for exclusion to ensure transparency and reproducibility.

### 2.5 Data extraction and charting

A standardised data charting form was developed to ensure systematic and consistent extraction of key information across all included studies. Ultimately, it supported a coherent synthesis and presentation of findings across the diverse body of literature. The predefined categories captured study characteristics (title, authors, year of publication, country), journal and study design, sample size, dental focus and dental setting, AI methodology, imaging system, gold standard reference, diagnostic accuracy measures, validation approaches, key findings, limitations and bias, study conclusions, and recommendations for research or practice. Data were extracted independently by two authors (SK & AG), with discrepancies resolved through discussion. Citations were managed in EndNote 21 and transferred to Microsoft Excel, where the extracted data were recorded for organisation and analysis.

### 2.6 Data analysis and synthesis

Following data extraction, the findings were collated and summarised to provide an overview of the included evidence. A descriptive numerical analysis, consistent with the JBI framework, was conducted to map key study characteristics. The analysis quantified the number and types of included studies, their geographical distribution, the AI models used, the intraoral imaging modalities, the validation methods, the gold standards, and the reported diagnostic performance metrics (e.g., sensitivity, specificity, AUC). This approach not only mapped the scope and distribution of existing evidence but also highlighted gaps in the literature, providing a foundation for the subsequent thematic synthesis. The descriptive analysis thus established a structured understanding of the evidence base, supporting the review’s objectives and informing practice, policy, and future research.

A narrative synthesis and thematic analysis were conducted to identify and integrate key patterns across the included studies, following Braun and Clarke’s six-phase thematic analysis framework to ensure transparency and rigour. ^33^

Included studies were reviewed in full and coded using a combined deductive–inductive approach, guided by the review objectives. Codes capturing methodological features, diagnostic performance, clinical relevance, validation strategies, and implementation barriers were organised into thematic categories. These themes were iteratively refined, supported by representative data, and presented through narrative synthesis alongside visual outputs (tables, matrices, heatmaps, and a thematic concept map). Coding and synthesis were performed manually using structured Excel tools, enabling integration of qualitative and quantitative insights with implications for research, practice, and policy.

## 3. Results

### 3.1 Selection of sources of evidence

**Figure 1.**
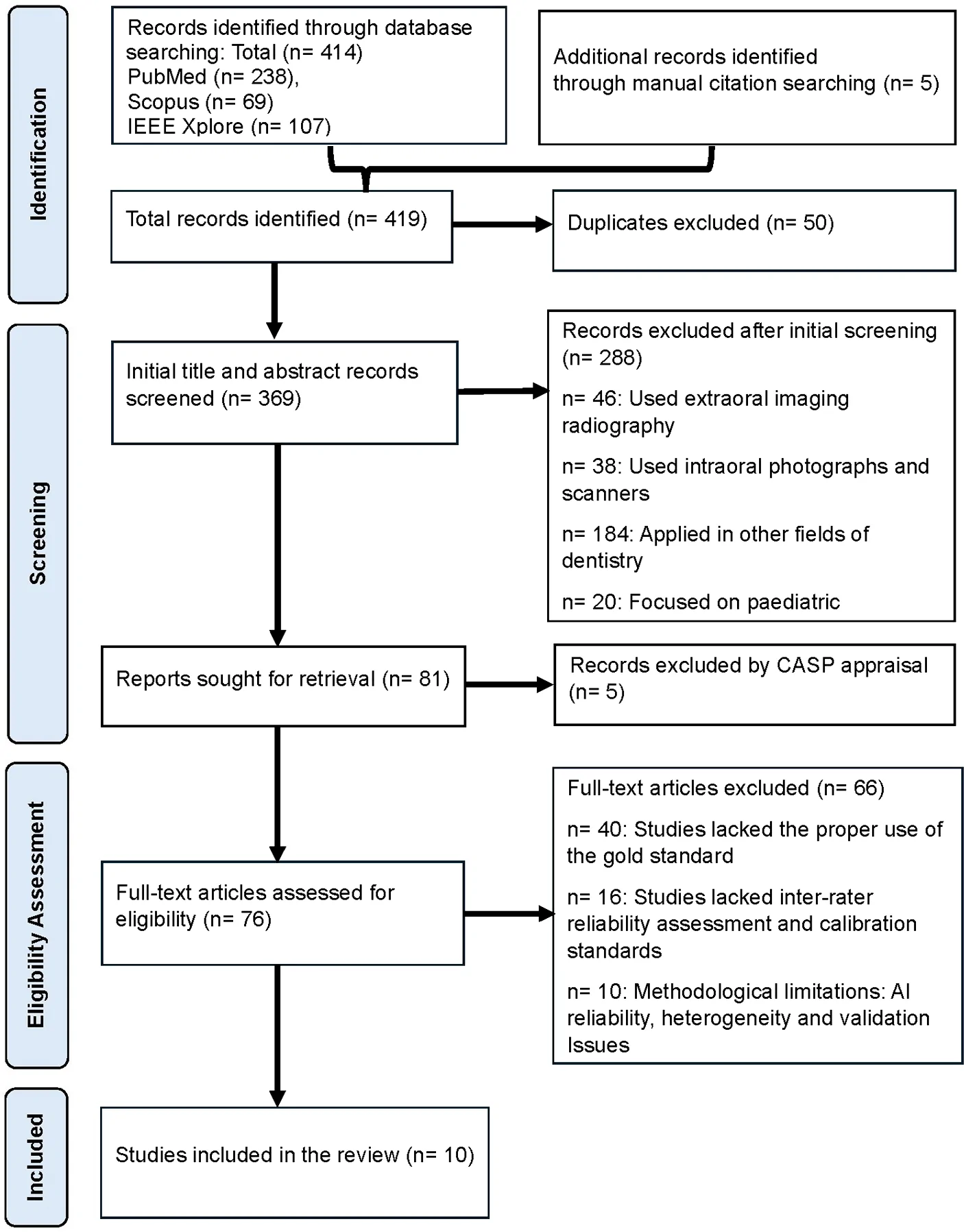
PRISMA flowchart for the scoping review selection of sources process.

The PRISMA-ScR flowchart ( Figure 1) illustrates the screening and selection process. Database searches retrieved 414 records (238 from PubMed, 69 from Scopus, 107 from IEEE Xplore) and five from manual searching, yielding 419 in total. After deduplication, 369 records remained for title and abstract screening, of which 288 were excluded for being irrelevant. Eighty-one articles progressed to full-text review with CASP appraisal. Seventy-one were excluded, five due to poor methodological quality and 66 because of the absence of a gold-standard comparator, inter-rater calibration, or adequate validation. Breakdown of reasons and numbers of studies excluded during title and abstract screening and full text assessment are provided in Table 1.

Ten studies met all criteria and were included in the final analysis. The included studies demonstrated generally high methodological quality, with clearly defined research aims, appropriate reference standards, robust statistical analyses, and relevant findings. The majority of the 10 studies met nearly all CASP criteria. The detailed quality appraisal results are provided in three Table 2 to Table 4, based on the study types. Table 2 presents diagnostic accuracy studies (n = 7), Table 3 includes randomised controlled trials (n = 2), and Table 4 presents the only cross-sectional study.

### 3.2 Characteristics of sources of evidence

**Figure 2.**
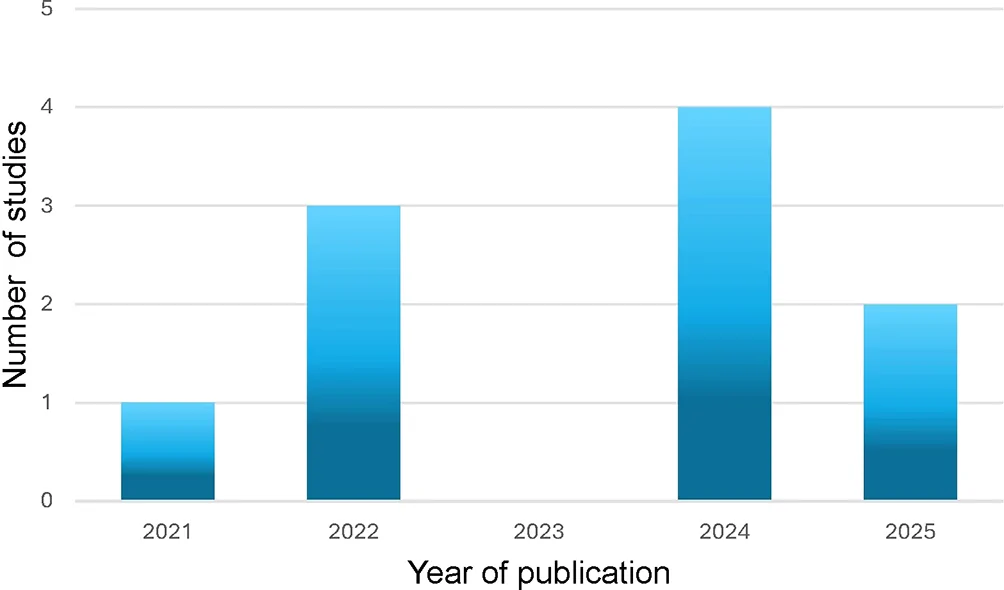
Distribution of studies by publication year.

The ten peer-reviewed studies included in the analysis were published between 2021 and 2025 ( Figure 2). The key characteristics of these studies are summarised in Table 5. The included studies represented diverse geographical settings: China (n = 4), Spain (n = 2), Germany (n = 2), the United Kingdom (n = 1), and a multinational collaboration between India and Saudi Arabia (n = 1). All were published in well-known dental and medical journals. ^34– 43^

**3.2.1 Study designs, settings and sample size**

The ten included studies comprised diagnostic accuracy studies (n = 4), in vitro diagnostic performance studies (n = 3), randomised controlled trials (n = 2), and one cross-sectional observational study. Most were conducted in laboratory or simulated environments using extracted human teeth, while others took place in university hospitals or private dental practices. ^34– 43^ The two RCTs directly examined AI’s impact on dentists’ diagnostic performance in both teaching and general practice settings, supporting AI’s potential role in clinical decision-making. ^38, 43^ Sample sizes ranged widely, from 171 proximal surfaces in extracted teeth to over 12,000 tooth-level images from clinical radiographs. Clinical datasets included adults aged 16–85 years, with examples such as García-Cañas et al. (n = 150 patients, 300 bitewings), ^40^ Lin et al. (n = 800 periapicals), ^41^ and Das et al. (n = 200 intraoral radiographs). ^38^ One study (Devlin et al.) uniquely explored AI’s role in education by involving 23 dentists in the interpretation of 24 bitewings. ^43^ While sample sizes supported both proof-of-concept and large-scale validation, detailed demographic reporting was generally absent.

**3.2.2 Diagnostic approaches and AI integration**

**Figure 3.**
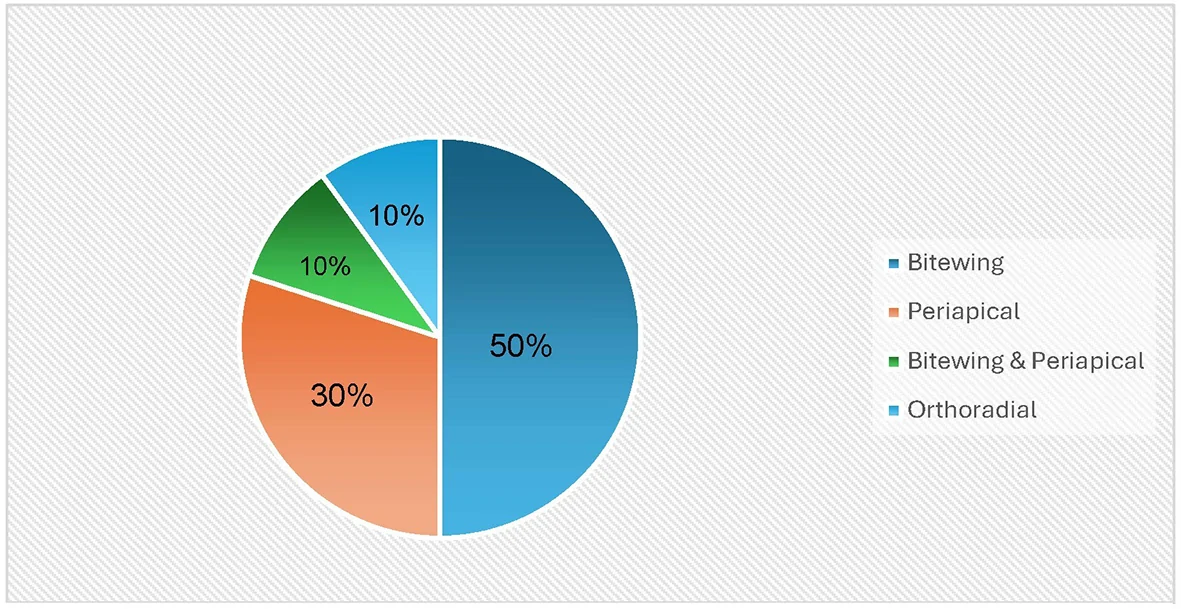
Distribution of radiograph types among included studies.

Most studies focused on detecting proximal carious lesions (n = 9), with one specifically addressing enamel-only caries. Some, such as Klein et al., targeted primary proximal lesions, while others (e.g., Ying et al., Chen et al.) examined both enamel and dentine involvement, and several proposed frameworks for grading lesion severity to improve benchmarking. All studies used intraoral radiographic imaging, most commonly digital bitewings (n = 5) and periapicals (n = 3), with one study combining both and another employing orthoradial radiographs. Acquisition techniques were generally standardised, though sensor brands were inconsistently reported ( Figure 3). ^34– 43^

AI methodologies were dominated by deep learning and convolutional neural networks, with tools such as Denti.AI and AssistDent integrated into radiographic interpretation. Object detection models (YOLOv5, DETR, Faster R-CNN) were applied for lesion localisation, while segmentation approaches (UNet, Trans-UNet, Segment Anything Model) supported precise lesion mapping. Some studies employed Grad-CAM to improve interpretability, and training strategies frequently included transfer learning, fine-tuning, and the use of pre-trained networks. Annotation aggregation techniques (e.g., majority voting, weighted voting, Dawid–Skene, MACE) were also used to enhance labelling reliability. ^34– 43^

**3.2.3 Gold standards and validation**

**Figure 4.**
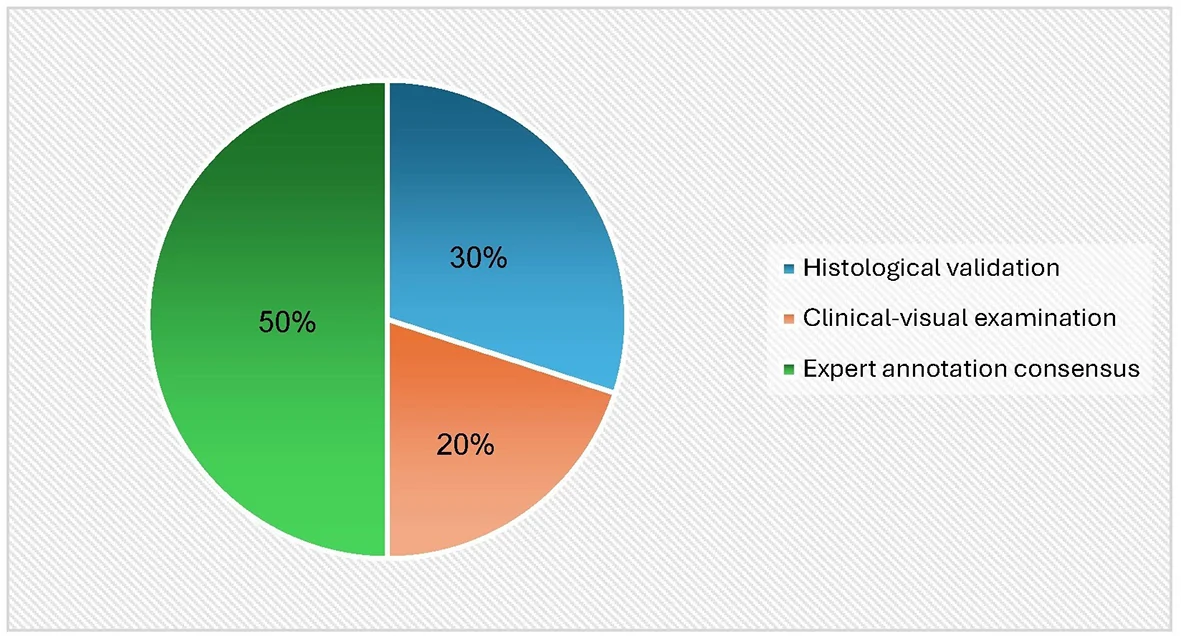
Gold standard validation methods were used in the included studies.

All studies employed a gold standard to evaluate AI accuracy in detecting dental caries from intraoral radiographs. Histological validation through thin-section microscopy was employed in three studies, while ICDAS-based clinical examination with cavity opening was used in two, and expert consensus with high calibration and inter-rater reliability was utilised in the remaining five ( Figure 4). While internal validation with expert panels, clinical examination, or blinded reviewers was most common, only one study conducted external benchmarking using a histology-based dataset. ^37^ Performance and agreement were assessed using statistical methods, including Cohen’s and Fleiss’ kappa, ICC, ROC curve and AUC analyses, as well as significance testing (Chi-square, McNemar’s, Wilcoxon, and Z-tests), with 95% confidence intervals typically reported. Some studies additionally employed Grad-CAM visualisation or benchmark thresholds to support interpretability and validate findings. ^34– 43^

### 3.3 Thematic analysis

**Figure 5.**
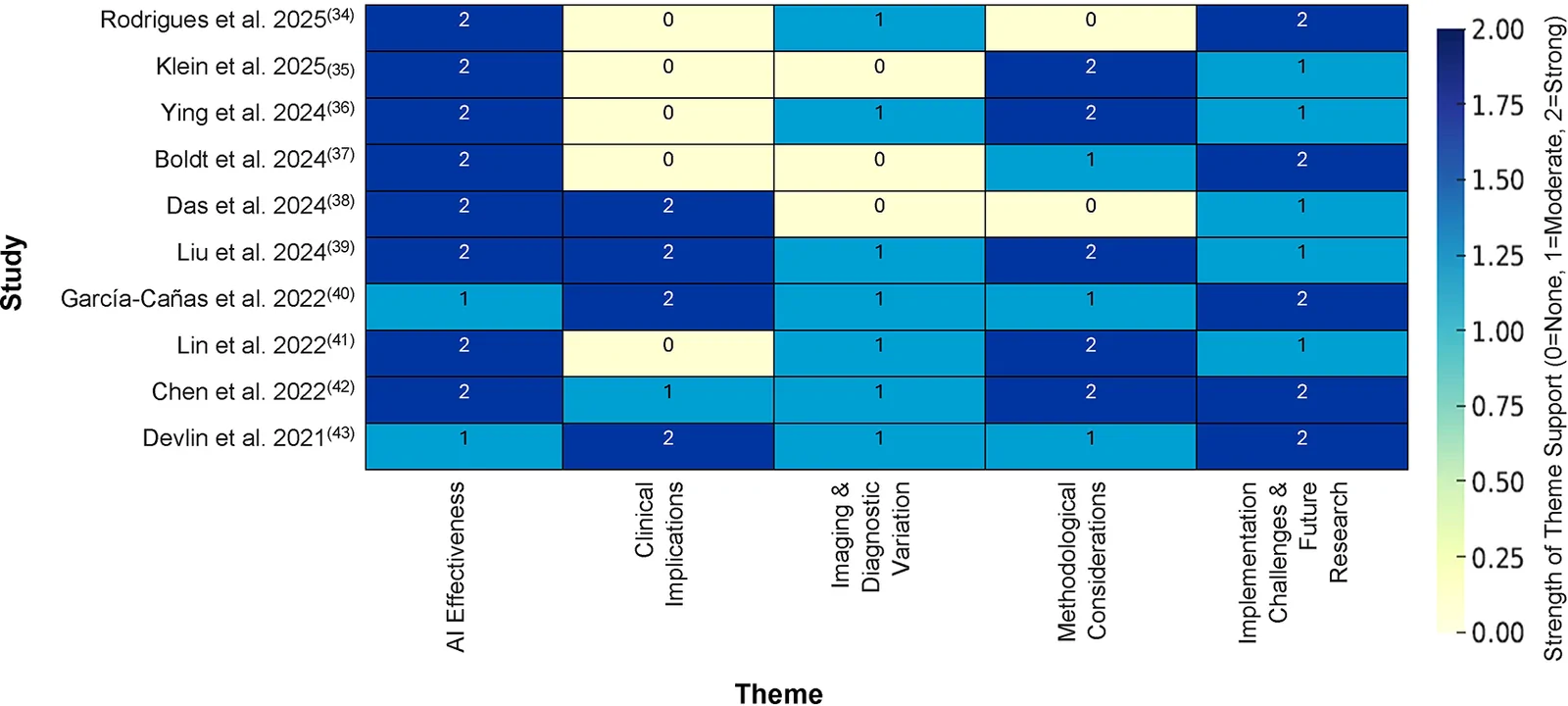
Theme coverage across included studies, stratified by strength of evidence.

**Figure 6.**
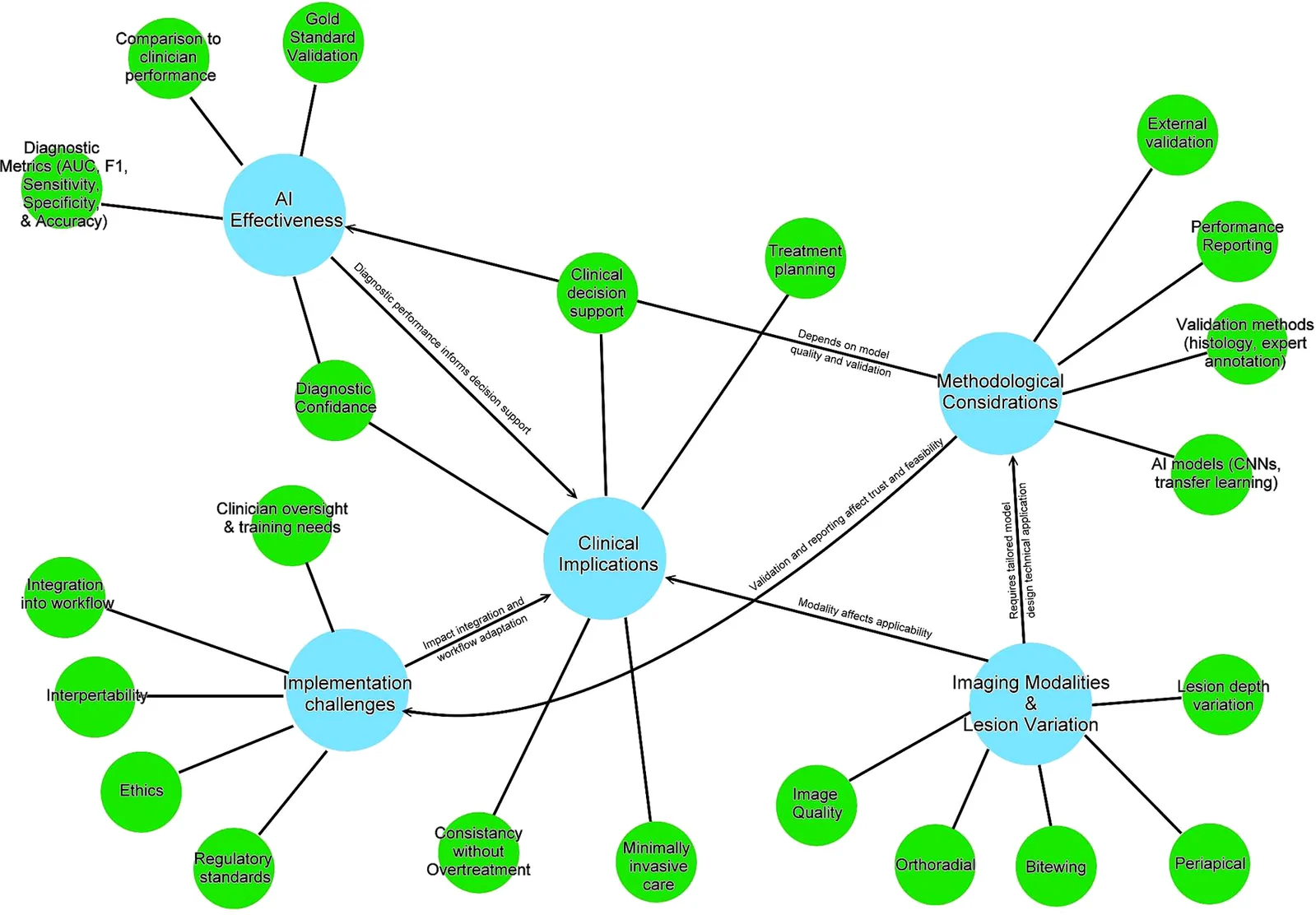
Thematic concept map: AI integration in dental caries detection.

The analysis identified five overarching themes that capture key insights on AI integration with intraoral radiographic imaging for caries detection in adults: (i) diagnostic accuracy performance, (ii) clinical relevance and implications, (iii) imaging-related factors, (iv) methodological considerations, and (v) recommendations for future integration. Each theme is described below with illustrative examples. The distribution of themes across studies and their primary outcomes is summarised in Table 6. Figure 5 presents a heatmap illustrating theme coverage and the strength of evidence, while Figure 6 displays the thematic concept map.

**3.3.1 Theme 1: AI effectiveness: Diagnostic accuracy and comparison with the gold standard**

Across the included studies, AI consistently demonstrated strong diagnostic potential, often matching or surpassing clinician performance, particularly in detecting early-stage and proximal caries. Reported sensitivity, specificity, accuracy, and F1-scores were generally higher for AI models than for human examiners. For instance, Devlin et al.’s study found that dentists using AI achieved 75.8% sensitivity and 85.4% specificity, compared with 44.3% and 96.3% without AI in detecting enamel-only caries. ^43^ In comparison, Das et al.’s RCT reported AI software performance of 88% sensitivity, 91% specificity, and 89% accuracy, exceeding human interpretation. ^38^ Chen et al.’s study also observed AI superiority over dental students, with 87% accuracy, 72% sensitivity, 93% specificity, and an F1-score of 0.74, compared with 82%, 47%, 94%, and 0.57, respectively. ^42^ Benchmarking against gold standards varied: histology-based studies reported the most stringent results, with specificity consistently >90% ^34, 35, 37^; ICDAS and cavity-opening studies, such as Liu et al. and García-Cañas et al., demonstrated accuracies of 82–88% and AUCs of 77–95% ^39, 40^; and expert consensus–based studies, such as Ying et al. and Lin et al., confirmed AI’s higher sensitivity than human eyes for both enamel and dentine caries ( *p* < 0.05). ^36, 41^ Collectively, these findings highlight AI’s consistent diagnostic reliability across diverse methodologies, settings, and comparator standards, underscoring its potential clinical utility in caries detection.

**3.3.2 Theme 2: Clinical implications and relevance: AI as a clinical decision support tool and impact on treatment planning**

AI integration with intraoral radiographs carries significant clinical implications, consistently enhancing diagnostic sensitivity, clinician confidence, and decision-making. Devlin et al.’s study demonstrated a 71% increase in sensitivity for enamel-only proximal caries when dentists used AI prompts (AssistDent), enabling earlier intervention and minimally invasive treatment planning. ^43^ Similarly, Chen et al.’s study demonstrated that AI enhanced the detection of early enamel and outer dentine lesions without substantially increasing false positives, particularly benefiting less experienced practitioners and highlighting its potential in dental education. ^42^ Across studies, AI was positioned as a decision-support tool rather than a substitute for clinicians, with the capacity to reduce overtreatment, support preventive care planning, and alleviate clinician workload through precise and timely detection of early lesions. ^34– 43^ Moreover, Devlin et al.’s study proposed that AI-supported sensitivity could serve as the basis for an audit standard for caries detection, underscoring AI’s transformative potential to promote evidence-based, patient-centred dental care. ^43^

**3.3.3 Theme 3: Imaging modalities and diagnostic variation by radiograph type and lesion severity**

Digital bitewing radiographs emerged as the predominant modality (n = 5), reflecting their widespread clinical use for detecting proximal caries. The remaining studies utilised periapical radiographs, with one combining bitewing and periapical images and another employing an orthoradial radiograph. Diagnostic performance varied according to lesion depth, severity, and radiograph type, with reduced accuracy frequently observed on images with tilted or low-quality radiographs. Studies by Liu et al. (2024) and Lin et al. (2022) highlighted how artefacts, anatomical variability, and positioning inconsistencies influenced outcomes, emphasising the importance of methodological refinement and improved standardisation of imaging and preprocessing. ^39, 41^ Several studies also reported that manual preprocessing tasks (e.g., image rotation or cropping) limited workflow efficiency, underscoring the need for automated solutions and more robust AI models capable of handling variations in real-world clinical imaging.

**3.3.4 Theme 4: Methodological considerations: AI model strategies, technical design, validation approaches, and limitations**

Substantial methodological diversity influenced the robustness and reliability of the included studies. A variety of convolutional neural networks (CNNs) were employed, including YOLOv5, Faster R-CNN, and ResNet variants, often enhanced with techniques such as edge extraction, transfer learning, and annotation aggregation strategies (e.g., MACE, Dawid–Skene) to optimise performance on small or imbalanced datasets. Validation approaches varied considerably: studies using histological ground truth provided the most objective benchmarks, while those relying on expert-labelled annotations introduced greater subjectivity and variability in results. Common limitations included the risk of overfitting, absence of multicentre validation, and reliance on imbalanced datasets, all of which restrict the generalisability of findings and underscore the need for more rigorous, standardised methodologies in future research. ^34– 43^

**3.3.5 Theme 5: Implementation challenges, recommendations for practical integration, and future research directions**

Implementation considerations featured prominently across the studies, with AI consistently framed as a supportive adjunct rather than an autonomous tool, reinforcing the principle that clinicians retain ultimate responsibility for diagnostic decisions. Central to effective integration were the development of interpretability mechanisms (e.g., Grad-CAM) to build clinician trust, alongside clear boundaries of clinical accountability, ethical safeguards, and regulatory clarity. Barriers to real-world adoption included the need for explainability, clinician training, and seamless incorporation of AI into established diagnostic frameworks such as ICDAS and ICCMS. To address these challenges, studies recommended comprehensive pilot testing, longitudinal and multi-centre validation, integration with risk-based caries management frameworks, and the use of AI for education and quality improvement feedback. ^34– 43^ Collectively, these recommendations emphasise the importance of establishing structured pathways to ensure the reliable, ethical, and effective clinical adoption of AI in caries detection.

## 4. Discussion

### 4.1 Interpretation of the main themes

Across the ten included studies, AI consistently demonstrated diagnostic performance comparable to, and in many cases exceeding, that of clinicians, particularly in detecting early-stage enamel and outer dentine caries. ^34– 43^ Metrics such as sensitivity, specificity, and AUC confirmed this trend. For instance, Das et al.’s study reported AI sensitivity and specificity above 88%, outperforming clinicians, ^38^ while Chen et al.’s study highlighted AI’s superior accuracy compared with dental students. ^42^ These findings align with broader systematic reviews, which show pooled CNN accuracies of 73–99% and sensitivities of 72–95%. ^44^ Nevertheless, performance varied considerably across AI model types, datasets, and lesion severity, with some models underperforming on subtle enamel lesions despite high specificity. ^34– 43^ This reinforces AI’s value as a diagnostic adjunct but highlights its current limitations for the most challenging lesion types.

Furthermore, it is important to emphasise that the diagnostic performance metrics reported in this scoping review primarily pertain to the detection of proximal caries using bitewing radiographs. Buccal and occlusal carious lesions - areas that are inherently more challenging to assess radiographically and are often underdiagnosed - were insufficiently represented across the included studies. ^34– 43^ Consequently, the generalisability of the reported AI performance outcomes to these lesion sites remains limited and warrants further investigation.

A significant source of variability was the type of gold standard used for diagnostic comparison. Studies employing histology-based validation provided the most objective benchmarks, with specificity consistently reported to exceed 90%. ^34, 35, 37^ In contrast, reliance on expert panel consensus annotations alone introduced subjectivity and moderate uncertainty, potentially inflating performance estimates. ^36, 41, 42^

Inconsistent reference standards undermine comparability, echoing concerns raised by the STARD guidelines on diagnostic research. ^45^ Furthermore, annotation protocols varied widely, ranging from single experts to multi-clinician panels with consensus methods, introducing heterogeneity in “ground truth” labelling and creating inherent limitations in validation accuracy. Since AI learns from the quality of its input data, variability in annotation reduces reproducibility and may embed systematic human errors into AI algorithms. ^35, 46^ This inconsistency in the gold standard, validation methods, and outcome measures used raises concerns about the generalisability and comparability of results across the evidence base.

Moreover, the absence of consistent and reliable gold-standard methods for evaluating the accuracy of AI in detecting dental caries also exist as a significant gap in the current literature across numerous studies, this critical limitation undermines the validity and reliability of reported AI performance metrics, resulting in a series of issues that impact clinical translation, regulatory approval, and meaningful comparison between different AI models. ^34, 35, 37^ The lack of robust reference standards is one of the most significant methodological challenges in contemporary dental AI research.

This review highlights significant clinical implications, particularly the role of AI as a decision-support tool. AI-enhanced radiographs improved clinicians’ sensitivity for early lesions, supported preventive interventions, and increased confidence in treatment planning. Devlin et al.’s study demonstrated a 71% increase in sensitivity for enamel-only lesions when clinicians utilised AI prompts. ^43^ In contrast, Chen et al.’s study reported improved performance with fewer false positives, particularly benefiting less experienced practitioners. ^42^ These outcomes align with evidence that AI augments diagnostic confidence, reduces variability, and facilitates minimally invasive dentistry. ^34, 39, 40, 42^ Importantly, AI was consistently positioned as an adjunct, not a replacement for clinical expertise, reinforcing its role in supporting evidence-based, patient-centred care.

Beyond diagnosis, AI demonstrated value in treatment planning, workflow efficiency, and education. The RCTs confirmed that clinicians supported by AI reduced false negatives and improved decision-making between preventive and operative strategies. ^38, 43^ Devlin et al.’s study also suggested AI could serve as an audit tool for caries detection. ^43^ This aligns with the broader literature; for instance, Pul et al.’s study highlighted its benefits for junior dentists, including improved diagnostic confidence and reduced overtreatment. ^47^ These findings suggest that AI may standardise diagnostic quality across different levels of experience, reducing disparities in dental care. However, evidence linking AI-supported diagnosis to long-term clinical outcomes (e.g., lesion progression, patient satisfaction) remains limited, highlighting an essential gap for future research.

Bitewing radiographs were the most widely used modality and consistently demonstrated higher diagnostic accuracy than periapical, particularly for proximal and early lesions. For instance, García-Cañas et al.’s study confirmed the superiority of bitewings for detecting enamel lesions. ^40^ In contrast, Lin et al.’s study reported lower sensitivity for periapical images due to angulation and anatomical overlap. ^41^ These findings align with those of Takahashi et al.’s study, who found that sensitivity for enamel caries was more than double in bitewings compared to periapical radiographs. ^48^ However, AI performance declined with low-quality, tilted, or artefact-affected images, often requiring manual preprocessing. This raises concerns about efficiency and standardisation, as real-world imaging rarely achieves the laboratory-quality standards. Hence, automating preprocessing and testing cross-modality generalisability remains a critical priority.

The methodological diversity across studies significantly influenced reported outcomes. Convolutional neural networks (CNN) architectures such as YOLOv5, ResNet, DETR, UNet, and SAM were employed, often enhanced with transfer learning, edge extraction, and aggregation strategies (e.g., MACE) to address dataset limitations. While some models achieved superior performance (e.g., YOLOv5 outperforming DETR and UNet), external benchmarking was rare.

A major barrier to the clinical translation of AI-based caries detection systems identified in this review is the persistent reliance on single-centre datasets. Most included studies relied primarily on internal validation methods, such as cross-validation or partitioning of a single-centre dataset. Although some investigations, such as Boldt et al. (2024), ^37^ strengthened methodological rigour through the development of histologically validated benchmarking datasets, robust external validation using independent, multicentre clinical data remains scarce. While internally validated models frequently report high diagnostic performance, the absence of external and multicentre evaluation limits confidence in their generalisability across diverse populations, imaging systems, and clinical workflows. ^51^ Consequently, the applicability of reported AI performance to routine clinical practice across heterogeneous settings remains uncertain. ^34– 43^ Practical barriers—including limited access to large, diverse datasets, logistical and financial challenges associated with multicentre collaboration, and proprietary constraints linked to commercial AI systems—likely contribute to this gap.

Similar concerns have been widely documented in the dental AI literature, where methodological reviews caution that reliance on internal validation may overestimate diagnostic performance, increase the risk of overfitting, and reduce reproducibility, particularly when model parameters and outcome definitions are inconsistently reported. ^51– 53^ For instance, the AI-Dentify study, which relied on internal cross-validation within a single cohort, and other recent single-centre bitewing investigations acknowledge restricted generalisability and call for multicentre validation. ^54^

Importantly, these methodological limitations carry significant regulatory and real-world deployment implications. Contemporary regulatory frameworks—including FDA, the UK's MHRA and international Good Machine Learning Practice (GMLP) guidance—increasingly require evidence of robust performance across multiple independent settings, emphasising the need for robust external validation using representative, independent datasets from sites distinct from those used in model development, as well as post-market performance monitoring to detect potential degradation or data drift. ^55, 56^

In parallel, broader methodological guidance, including recommendations from the FUTURE-AI Consortium, ^49^ highlights that transparent reporting and rigorous external and multicentre validation are essential prerequisites for safe and effective clinical implementation. Collectively, this evolving regulatory and methodological landscape underscores the urgent need for standardised external and multicentre validation frameworks to strengthen the reliability, generalisability, regulatory readiness, and clinical utility of dental AI systems in real-world practice.

Lastly, practical integration faces significant barriers. While AI shows strong diagnostic promise, studies consistently emphasised its supportive role under clinician oversight. Barriers include a lack of standardised interpretability tools, unclear regulatory pathways, ethical considerations around patient consent, and infrastructural demands in smaller practices. Tools like Grad-CAM were proposed to enhance trust by visualising AI reasoning; however, real-world deployment remains limited. Furthermore, integrating AI into established frameworks, such as ICDAS and ICCMS, was recommended to align AI outputs with risk-based caries management; however, evidence of feasibility remains limited. Hence, pilot studies and clinician training in AI literacy are essential to ensure responsible adoption, prevent over-reliance, and establish robust regulatory frameworks.

### 4.2 Strengths and limitations

This scoping review has several notable strengths. The rigorous methodological design ensured transparency, reproducibility, and comprehensiveness throughout study identification, selection, data charting, and synthesis, which enhanced the robustness and reliability of the findings. The inclusion of both technical and clinical studies provided broad coverage, ranging from in vitro validations of extracted teeth to in vivo evaluations in practical settings. This breadth offers a holistic understanding of AI integration into intraoral radiographic imaging for caries detection, bridging technical innovation with clinical relevance. Additionally, thematic synthesis enabled effective mapping across diverse study designs, methodologies, and outcomes, providing cross-disciplinary insights into areas of consensus, divergence, research gaps, and practical implications for clinical care. Additionally, all included studies were recent peer-reviewed publications (2021–2025), ensuring that the findings reflect the most current evidence on AI applications, validation standards, and emerging diagnostic trends.

In contrast, several limitations should also be acknowledged. Restriction to English-language publications may have introduced language and publication bias, potentially excluding relevant evidence. The small number of included studies (n = 10) and their methodological heterogeneity-particularly in definitions of gold standards, outcome measures, and study designs-limited comparability and prevented the conduct of a meta-analysis. Furthermore, most studies were preclinical or early diagnostic accuracy trials, with few addressing patient-centred outcomes such as lesion progression, treatment effectiveness, or long-term impacts on caries management, thereby limiting clinical relevance. Furthermore, small sample sizes, reliance on single-centre datasets, and lack of multicentre validation further restrict generalisability. Addiotionally, potential bias may have arisen from the use of overlapping datasets or developer involvement in multiple studies, which could inflate diagnostic accuracy estimates. These limitations highlight the need for independent, multicentre studies employing standardised methods to strengthen the evidence base for AI-assisted dental diagnostics.

Finally, a key limitation of this review, which is also the limitation of using intra-oral x-rays for diagnosis of dental caries, is that the reported diagnostic performance metrics primarily pertain to proximal caries detection using bitewing radiographs. Buccal and occlusal caries were insufficiently represented in the included studies. This imbalance limits the generalisability of the findings and underscores the need for future research to evaluate AI performance across a broader range of anatomical lesion sites and clinical scenarios.

### 4.3 Implications for clinical practice

The findings of this scoping review highlight essential implications for dental practice. The integration of AI into intraoral radiographic diagnostics supports minimally invasive dentistry by enabling the earlier and more accurate detection of carious lesions, particularly at incipient stages. This allows clinicians to prioritise timely preventive interventions over invasive restorative treatments. AI-assisted diagnostics also offer the potential to standardise clinical decision-making, reducing inter-examiner variability and enhancing consistency in patient care across practitioners with differing levels of experience.

Successful adoption of AI in practice, however, requires robust clinician training and oversight to ensure that these tools are used as adjuncts to, rather than replacements for, clinical judgment. Embedding AI literacy into dental education and continuing professional development is therefore essential. Training should equip clinicians to interpret AI outputs critically, recognise potential biases, and address the ethical and practical challenges of AI-assisted care. Such educational investment will be pivotal to optimising patient outcomes, fostering clinician confidence, and ensuring responsible integration of AI into routine dental diagnostics.

### 4.4 Recommendations for future research

This review highlights key priorities for advancing AI integration into dental caries detection. Standardising validation protocols is crucial; future research should include robust, universally accepted benchmarks such as histological gold standards, multicentre validation, and longitudinal follow-up in clinical settings. Such consistency would enhance comparability across studies and strengthen conclusions about diagnostic accuracy. Furthermore, prospective real-world clinical trials are necessary to assess AI systems in routine practice, considering feasibility, performance, and impacts across diverse populations, imaging techniques, and workflows to ensure generalisability.

Beyond diagnostic accuracy, future research should evaluate cost-effectiveness, usability, clinician and patient acceptability, and patient-centred outcomes, including lesion progression, treatment effectiveness, and quality of life. Exploring patient trust and satisfaction with AI-driven diagnostics represents a remarkably underexplored dimension. Comparative analyses of various AI architectures are also necessary to determine the most effective ones for radiographic caries detection, with semantic segmentation and explainable AI methods, such as Grad-CAM, showing potential. Research into AI as a primary screening tool or as an adjunct, supporting dental professionals in capturing and interpreting radiographs with less reliance on direct supervision, could guide its integration into diagnostic pathways, enhancing workflow efficiency while maintaining diagnostic standards.

## 5. Conclusions

This scoping review highlights the substantial diagnostic potential of AI integrated with intraoral digital radiographic imaging systems for detecting dental caries in adults. AI has shown promise, particularly in identifying early-stage and proximal carious lesions, thereby supporting minimally invasive and preventive treatment strategies.

These findings should be interpreted within the specific anatomical scope of the included evidence. The reported diagnostic performance metrics and supporting data predominantly relate to proximal caries detection on bitewing radiographs rather than to dental caries in general. Consequently, the performance outcomes of the evaluated AI systems cannot be directly extrapolated to buccal or occlusal lesions, which were underrepresented in the current evidence base and require further targeted investigation.

Despite encouraging diagnostic accuracy, the full realisation of AI’s clinical potential in dental radiology depends on overcoming several key limitations. These include the lack of standardised external and multi-centre validation across diverse populations and clinical settings, and the need for comprehensive clinician training to ensure accurate interpretation of AI outputs and foster professional trust. AI should therefore be positioned as a supportive tool that augments, rather than replaces, clinical expertise. Adopting this collaborative model, where AI enhances diagnostic precision, standardises care, and enables earlier interventions, offers a pathway to advancing minimally invasive dentistry and improving patient outcomes. Ultimately, the responsible integration of AI into intraoral radiographic diagnostics represents a transformative step towards more accurate, efficient, and patient-centred dental care.

## Data Availability

All supplementary files can be found in the external repository: “Data set and Prisma checklist-Artificial Intelligence Integrated with Intra-oral Digital Imaging in Dental Caries Detection, Treatment Planning, and Clinical Decision-Making: A Scoping Review” https://qmro.qmul.ac.uk/xmlui/handle/123456789/113231. Data are available under the terms of the Creative Commons Zero “No rights reserved” data waiver (CC0 1.0 Public domain dedication).
