# Supplementary material for: Artificial Intelligence Integrated with Intraoral Digital Imaging in Dental Caries Detection, Treatment Planning, and Clinical Decision-Making: A Scoping Review
Source: F1000Res. 2026 Mar 5;14:1328. Originally published 2025 Nov 27. [Version 2] doi: 10.12688/f1000research.172671.2 (PMC13280539; doi:10.12688/f1000research.172671.2)
Supplement: Peer review history

# Reviewer report: Reviewer response for version 2

**DOI:** 10.5256/f1000research.197199.r449985

**Published:** 2026-08-17

**Authors:** Omid Panahi

**Affiliations:** 1 Centro Escolar University, Manila, Metro Manila, Philippines

This manuscript provides a timely bibliometric review of emerging technologies in medical device development, with particular emphasis on Artificial Intelligence (AI), the Internet of Medical Things (IoMT), Augmented Reality (AR), Big Data, cybersecurity, and embedded systems. The authors analyzed publications indexed in Scopus and Web of Science from 2010 to 2024 and used Bibliometrix/Biblioshiny and VOSviewer to identify publication trends, geographical contributions, keyword patterns, and major research clusters. The manuscript addresses an important and rapidly developing area and provides a useful overview of the research landscape. The discussion has also been expanded in the revised version to address ethical, clinical, privacy, bias, and user-training considerations. However, several issues remain before the review can be considered fully comprehensive. In particular, the manuscript remains predominantly bibliometric, while its title suggests a broader critical review of **trends, challenges, and future directions in the development of medical devices**. Consequently, some technological and clinical claims are broader than the evidence generated by the bibliometric analysis. Previous reviewers have identified a similar mismatch between the title and the actual scope of the analysis.

 1. Comprehensiveness of the topic — **Partly**

 The manuscript covers the principal technologies identified in its research questions, including AI, IoMT, AR/VR, Big Data, cybersecurity, and embedded systems. It also addresses important issues such as privacy, interoperability, infrastructure, ethics, and clinical adoption.

 Nevertheless, the literature discussion is not yet fully comprehensive in the context of the current research landscape.

 The main limitation is that the manuscript relies heavily on bibliometric indicators to characterize technological development. Publication counts and keyword co-occurrence are useful for identifying research activity, but they do not provide a sufficiently detailed critical assessment of how these technologies are actually being implemented in medical devices.

 The authors should strengthen the review by:

- adding representative real-world examples or case studies of AI-enabled, IoMT-enabled, AR/VR-enabled, and cybersecurity-focused medical devices;
- discussing technological maturity and implementation barriers;
- expanding the discussion of interoperability and regulatory requirements;
- addressing contemporary cybersecurity threats affecting connected and AI-enabled medical devices;
- discussing privacy-preserving approaches such as federated learning and edge computing where relevant;
- more clearly identifying the specific research gap addressed by this review compared with previous reviews.

These points are particularly important because the current manuscript claims to address "trends, challenges, and future directions," while much of the results section remains focused on bibliometric mapping.

 2. Accuracy and citation support — **Partly**

 Most major factual statements are accompanied by citations, and the manuscript uses references throughout its technology-specific sections. For example, claims concerning IoMT, AR/VR, Big Data, and cybersecurity are generally referenced.

 However, some statements are broader than the cited evidence appears to justify.

 For example, the conclusion states that advanced technologies are increasingly being adopted "to improve healthcare outcomes" and that they "hold significant potential to improve patient outcomes, modernise clinical workflows, and accelerate personalized medicine."

 These statements may be reasonable in the broader literature, but the **bibliometric analysis itself cannot demonstrate clinical effectiveness or improved patient outcomes**. The authors should therefore either:

1. provide specific contemporary references demonstrating these clinical effects, or
2. rephrase these statements to indicate that the technologies have *potential* or that these outcomes are *reported/being investigated in the literature*, rather than implying that the present bibliometric study established them.

Similarly, statements about countries such as Morocco making "significant strides" should be interpreted carefully because bibliometric publication output does not necessarily establish technological leadership, clinical implementation, or practical impact.

 3. Accessibility of language — **Partly**

 The manuscript is generally understandable, and the overall structure is logical. The major concepts are accessible to readers from healthcare, engineering, and digital-health backgrounds.

 However, there are recurring grammatical and stylistic problems that reduce readability and academic precision. Some sentences are overly general or use informal formulations. Previous reviewer assessment also identified "minor grammatical inconsistencies and informal phrasing."

 I recommend a careful professional English-language revision, particularly for:

- article usage and singular/plural agreement;
- verb tense and subject–verb agreement;
- unnecessarily long sentences;
- repetitive descriptions of Scopus and Web of Science;
- inconsistent terminology such as IoT versus IoMT;
- wording that implies causality where only association or research interest has been demonstrated.

The authors should also reduce repetitive statements such as "transforming healthcare," "improving patient outcomes," and "revolutionizing healthcare" unless these claims are specifically supported.

 4. Conclusions — **Partly**

 The overall conclusions are directionally consistent with the bibliometric findings. The identified clusters—AI/AR, IoT/cybersecurity, and embedded systems—are supported by the analysis.

 However, the conclusions still occasionally extend beyond the evidence generated by the study.

 The bibliometric results demonstrate **research trends and thematic associations**, rather than proving that these technologies improve clinical outcomes or that they have already been successfully adopted across healthcare systems.

 The conclusion should therefore be revised to distinguish clearly between:

- findings directly demonstrated by the bibliometric analysis;
- implications suggested by the broader literature;
- potential future applications.

This is particularly important for statements concerning patient outcomes, personalized medicine, clinical workflow improvement, and technological leadership of particular countries.

 Essential revisions for scientific soundness

 The following points should be addressed before the manuscript can be considered fully scientifically sound:

**1. Strengthen the critical literature synthesis.**

 The authors should expand beyond bibliometric indicators and provide a more substantive discussion of the technological evolution and real-world application of AI, IoMT, AR/VR, Big Data, and cybersecurity in medical devices.

**2. Correct or qualify unsupported factual claims.**

 Claims concerning clinical effectiveness, patient outcomes, technological leadership, or successful implementation should either be supported with appropriate contemporary references or appropriately qualified.

**3. Align the conclusions with the evidence.**

 The conclusions should explicitly acknowledge that the study maps research activity and thematic trends rather than directly evaluating clinical efficacy or technological performance.

**4. Improve language and readability.**

 A thorough English-language edit is recommended to remove grammatical inconsistencies, repetition, and overly broad claims.

**5. Clarify the novelty and research gap.**

 The authors should explicitly explain what this review contributes beyond existing reviews of emerging technologies in medical devices.

**Overall assessment**

 The manuscript addresses an important and current topic and provides a useful bibliometric overview of research involving emerging technologies in medical devices. The use of two major bibliographic databases and established bibliometric tools provides a reasonable foundation for the analysis.

 However, the manuscript would benefit from a more comprehensive and critical engagement with the current literature, stronger citation support for broad factual claims, improved academic language, and more cautious conclusions. The principal issue is the distinction between **mapping the research literature** and **demonstrating technological or clinical impact**.

 I therefore recommend **major revision**. The requested changes are specific and achievable, and addressing them would substantially improve the scientific rigor, clarity, and usefulness of the review.

Is the review written in accessible language?

Partly

Are all factual statements correct and adequately supported by citations?

Partly

Are the conclusions drawn appropriate in the context of the current research literature?

Partly

Is the topic of the review discussed comprehensively in the context of the current literature?

Partly

Reviewer Expertise:

Artificial Intelligence in Healthcare; Digital Health Technologies; Internet of Medical Things (IoMT); Biomedical Engineering; Medical Device Innovation; Machine Learning Applications in Healthcare; Healthcare Data Analytics; Cybersecurity in Healthcare; Bibliometric Analysis.

I confirm that I have read this submission and believe that I have an appropriate level of expertise to state that I do not consider it to be of an acceptable scientific standard, for reasons outlined above.

# Reviewer report: Reviewer response for version 2

**DOI:** 10.5256/f1000research.197199.r485638

**Published:** 2026-06-18

**Authors:** Pankaj Kukreja

**Affiliations:** 1 Al Baha University, Al Bahah, Al Bahah Province, Saudi Arabia

The study is well written, it is methodologically robust, transparent, and well aligned with JBI and PRISMA‑ScR standards. The search strategy and eligibility criteria are particularly strong.

 I have a few points which I feel authors should have addressed-

 1. AI involvement in critical appraisal is unconventional and may raise questions about reliability. It is unclear how the AI was “trained and calibrated.”

 2. Authors state in eligibility criteria that 'reviews of these original studies were included.' Were review articles used only for citation chasing, were they included in data extraction, and how was overlapping data handled?

Is the review written in accessible language?

Yes

Are all factual statements correct and adequately supported by citations?

Yes

Are the conclusions drawn appropriate in the context of the current research literature?

Yes

Is the topic of the review discussed comprehensively in the context of the current literature?

Yes

Reviewer Expertise:

Oral and maxillofacial surgery, Dental Education, AI in medical education.

I confirm that I have read this submission and believe that I have an appropriate level of expertise to confirm that it is of an acceptable scientific standard.

# Reviewer report: Reviewer response for version 1

**DOI:** 10.5256/f1000research.190413.r449986

**Published:** 2026-02-03

**Authors:** Patricio Melendez Rojas

**Affiliations:** 1 Universidad de Valparaíso, Valparaíso, Chile

The scoping review by Kayali et al. (2025) provides a systematic mapping of artificial intelligence (AI) applications in dental caries detection, utilizing the Joanna Briggs Institute (JBI) framework and PRISMA-ScR guidelines to ensure methodological transparency. While the study successfully synthesizes 10 high-quality investigations published between 2021 and 2025, its scope is mainly limited to proximal caries, despite the title suggesting broader coverage of all dental caries. Factual accuracy is well supported by primary sources, which indicate that AI models—particularly those based on deep learning—frequently achieve superior diagnostic performance compared with human clinicians.

 The following recommendations are proposed to improve the scientific rigor and accessibility of the article:

1. **Enhance Technical Accessibility**: Explicitly define diagnostic metrics such as sensitivity, specificity, accuracy, precision, **AUC**, **F1-score**, **PPV**, and **NPV** upon their first mention in the text to ensure the content is accessible to general dental practitioners.
2. **Clarify Anatomical Scope**: Could you explain in the conclusions that the reported data and AI performance metrics relate specifically to proximal caries? The authors should recognize that buccal and occlusal caries are often underdiagnosed through radiographic methods and were not adequately represented in the studies included.
3. **Standardize Reporting of Reference Methods**: Incorporate a dedicated column in the summary tables to identify the specific **gold standard** used in each study, such as histological validation or expert consensus.
4. **Expand Discussion on Implementation Barriers**: Provide a more detailed analysis of the current lack of **external and multicentre validation** in dental AI research. Just to let you know, the reliance on single-centre datasets remains a primary obstacle to the clinical generalizability and regulatory approval of these diagnostic tools.
5. **Streamline and Decompartmentalize Table 3**: Given that Table 3 is exceptionally dense and spans seven consecutive pages, it is recommended to reorganize the data into smaller, thematic sub-tables to reduce cognitive load for the reader. The authors should consider separating **Study Characteristics** (e.g., Design, Country, and Imaging System) from **Diagnostic Performance and Outcomes** (e.g., Accuracy Measures, Gold Standards, and Key Findings). 
6. **Full Transparency of Search Strings**: Although the authors describe the conceptual framework of the literature search—including five thematic groups and the use of Boolean operators —the actual, reproducible search strings are currently relegated to an external data repository. For a scoping review following **PRISMA-ScR** guidelines, it is a best practice to include the complete, verbatim search query for at least one major database (e.g., PubMed) directly within the manuscript or as a formal appendix. 
7. **Consolidate Quality Appraisal Tables**: Tables 1.1, 1.2, and 1.3 are highly repetitive, with the majority of studies scoring "Yes" across nearly all **CASP** criteria. To improve flow, these detailed checklists should be moved to the **Annexes**, with the main text providing only a concise narrative summary of the high methodological quality observed across the sample.

Is the review written in accessible language?

Partly

Are all factual statements correct and adequately supported by citations?

Yes

Are the conclusions drawn appropriate in the context of the current research literature?

Yes

Is the topic of the review discussed comprehensively in the context of the current literature?

Partly

Reviewer Expertise:

Dento-maxillofacial radiology. AI applications in Health.

I confirm that I have read this submission and believe that I have an appropriate level of expertise to confirm that it is of an acceptable scientific standard, however I have significant reservations, as outlined above.

# Response

**Published:** 2026-02-26

**Authors:** Ali Golkari

**Affiliations:** Institute of Dentistry, Queen Mary University of London, London, England, UK

The authors sincerely thank the respected reviewer for accepting the peer-review and for the time they have spent to go through the manuscript in detail and provide us with such a valuable and constructive comment. The authors have done their best to amend the manuscript based on the reviewer’s comments and hope the changes are acceptable. However, any further comment or feedback is welcome.

 Please see below a point by point response:

**Comment 1: Enhance Technical Accessibility**

 Thank you for this valuable suggestion. We have now explicitly defined all diagnostic performance metrics at their first occurrence in the manuscript's methods section to enhance accessibility for general dental practitioners and non-technical readers.

**Comment 2: Clarify Anatomical Scope**

 We thank the reviewer for highlighting this important limitation. We have revised the Discussion, Limitations and Conclusions sections to explicitly state that the reported diagnostic performance metrics primarily relate to proximal caries detection and that buccal and occlusal lesions were inadequately represented in the included studies.

**Comment 3: Standardize Reporting of Reference Methods**

 We thank the reviewer for this helpful suggestion. We would like to clarify that Table 3 already includes a dedicated column specifying the reference standard (gold standard) used in each study, such as histological validation or expert consensus.

**Comment 4: Expand Discussion on Implementation Barriers**

 Thank you for this important observation. We have expanded the Discussion section to provide a more detailed analysis of reliance on single-centre datasets, the lack of external and multicentre validation in dental AI research, and their implications for clinical generalisability and regulatory approval.

**Comment 5: Streamline and Decompartmentalize Table 3**

 We thank the reviewer for this thoughtful suggestion and appreciate the concern regarding the density of this table. While we carefully considered reorganising the table into separate thematic sub-tables, we found that study characteristics, reference standards, and diagnostic performance outcomes are closely interrelated and are most meaningfully interpreted together. Separating these elements would risk fragmenting the key contextual information required to interpret reported accuracy measures appropriately within each study’s methodological framework.

**Comment 6: Full Transparency of Search Strings**

 We thank the reviewer for this recommendation. In accordance with PRISMA-ScR best practices, we have now included the complete, verbatim PubMed search strategy in the Methods to ensure full transparency and reproducibility.

**Comment 7: Consolidate Quality Appraisal Tables**

 We agree with this suggestion. More details as a concise narrative summary of the overall quality appraisal in now included in the result section. The CASP appraisal tables have originally uploaded as appendices when submitting the paper. The journal brought them into the main text. We will ask the production team to see if they can move them to appendices.

# Reviewer report: Reviewer response for version 1

**DOI:** 10.5256/f1000research.190413.r437139

**Published:** 2025-12-26

**Authors:** Xin Hui Yeo

**Affiliations:** 1 Bart´s Health NHS Trust, England, UK

A comprehensive review and thorough methodology, well done to the authors. A few comments for consideration:

 - It would be useful to define sensitivity, specificity, accuracy, precision, AUC, F1 score, PPC and NPV when first mentioned in the manuscript and then refer back to them if any studies reported those data.

 - The study's aim/concept is dental caries detection by AI and intraoral imaging. Dental caries can involve any tooth surfaces but only proximal caries has been reported. Is buccal or occlusal caries included in the inclusion criteria? Buccal caries can be more challenging to diagnosed on radiograph due to overlapping with the pulp chamber, resulting in under-diagnosis. If buccal caries is not part of the review or has not been reported by other primary studies, then perhaps consider adding this to the limitations and adjusting the conclusion to specify supporting data relevant to proximal caries instead of dental caries in general.

 - For table 3, it would be easier for the reader to have a column for gold standard reference used in each study (e.g. histology or expert consensus) for what the AI methodology was compared against.

Is the review written in accessible language?

Yes

Are all factual statements correct and adequately supported by citations?

Yes

Are the conclusions drawn appropriate in the context of the current research literature?

Yes

Is the topic of the review discussed comprehensively in the context of the current literature?

Yes

Reviewer Expertise:

review methodology, implant dentistry, digital dentistry

I confirm that I have read this submission and believe that I have an appropriate level of expertise to confirm that it is of an acceptable scientific standard.

# Response

**Published:** 2026-02-26

**Authors:** Ali Golkari

**Affiliations:** Institute of Dentistry, Queen Mary University of London, London, England, UK

We sincerely thank the reviewer for these insightful and constructive comments. Please see below a point by point response to the comments.

**1. Definition of diagnostic metrics:**

 In response to this suggestion, we have now explicitly defined all diagnostic performance metrics (sensitivity, specificity, accuracy, precision, positive predictive value [PPV], negative predictive value [NPV], F1-score, and area under the curve [AUC]) at their first occurrence in the Methods section. Subsequent references to these metrics in the Results section now refer back to these definitions to enhance clarity and accessibility for general dental practitioners and non-technical readers.

**2. Clarification of anatomical scope (proximal vs. buccal/occlusal caries)**

 We appreciate this important observation. Although the inclusion criteria did not restrict caries detection to a specific anatomical surface, the primary studies meeting eligibility criteria predominantly evaluated proximal caries using bitewing radiographs. Buccal and occlusal lesions were not adequately represented in the included evidence base.

**To address this, we have:**

 1. Clarified in the Discussion that the reported AI performance metrics relate primarily to proximal caries detection.

 2. Added a statement to the Limitations section acknowledging that buccal and occlusal caries—particularly buccal lesions, which may be underdiagnosed radiographically due to anatomical overlap—were underrepresented.

 3. Revised the Conclusion to specify that the supporting diagnostic performance data relate to proximal caries rather than dental caries in general.

 We believe these revisions improve the precision, transparency, and clinical interpretation of the findings. However, we greatly appreciate any further comment that can help improve the quality of this manuscript.
